# Informed consent in cancer clinical drug trials in China: a narrative literature review of the past 20 years

**DOI:** 10.1186/s13063-023-07482-y

**Published:** 2023-07-07

**Authors:** Xing Liu, Xiaoran Lu, Wei Zhou, Jessica Hahne, Kaveh Khoshnood, Xiaoting Shi, Yuqiong Zhong, Xiaomin Wang

**Affiliations:** 1 Medical Ethics Committee, Xiangya Hospital of Central South University, 87 Xiangya Road, Changsha, 410008 Hunan People’s Republic of China; 2 School of Humanities, Central South University, Changsha, 410075 Hunan People’s Republic of China; 3 School of Public Administration, Hunan University, Changsha, 410023 Hunan People’s Republic of China; 4 Department of Psychological & Brain Sciences, Washington University in St. Louis, St. Louis, MO USA; 5 Yale School of Public Health, Yale University, 60 College Street, New Haven, CT 06520 USA; 6 Department of Environmental Health Sciences, Yale School of Public Health, 60 College St, New Haven, CT 06520 USA; 7 Center for Clinical Pharmacology, The Third Xiangya Hospital, Central South University, Changsha, 410013 Hunan People’s Republic of China

**Keywords:** Cancer drugs, Clinical trials, Research ethics, Informed consent, Narrative review

## Abstract

**Background:**

Although the number of cancer clinical drug trials is increasing rapidly in China, issues concerning informed consent in this research context are understudied. By performing a narrative literature review, we aim to describe the current situation and identify the most salient challenges affecting informed consent in cancer clinical drug trials among adult patients in China since 2000.

**Methods:**

We searched Web of Science (WOS), PubMed, Scopus, EMBASE, the Cochrane Library databases, China National Knowledge Infrastructure (CNKI), China Biomedical Literature Database on Disc (CBMdisc), Chinese Scientific Journals Fulltext Database (CQVIP), and WANFANG Data to identify relevant publications since 2000. Data were extracted by three reviewers on six items pertaining to study type, theme, and challenges.

**Results:**

We identified 37 unique manuscripts, from which 19 full texts were obtained and six were included in the review. All six studies were published in Chinese journals, and the publication years of the majority (five out of six) of the studies were 2015 or later. The authors of the six studies were all from clinical departments or ethical review committees at five hospitals in China. All of the included publications were descriptive studies. Publications reported challenges related to the following aspects of informed consent: information disclosure, patient understanding, voluntariness, authorization, and procedural steps.

**Conclusion:**

Based on our analysis of publications over the past two decades, there are currently frequent challenges related to various aspects of informed consent in cancer clinical drug trials in China. Furthermore, only a limited number of high-quality research studies on informed consent in cancer clinical drug trials in China are available to date. Efforts toward improvement of informed consent practice, in the form of guidelines or further regulations in China, should draw on both experience from other countries and high-quality local evidence.

**Supplementary Information:**

The online version contains supplementary material available at 10.1186/s13063-023-07482-y.

## Background

Implementation of clinical trials is vital for continuing to develop more effective treatments for cancer, one of the leading causes of death on a global level [1]. An estimated 19.29 million new cancer cases occurred worldwide in 2020, including 4.57 million new cases and 3 million cancer-related deaths in China [2]. China ranks first in the world for both incidence of cancer (accounting for 23.7% of new global cases) and cancer deaths (accounting for 30% of all cancer deaths) [2]. Rates of new cancer cases and deaths in China have been increasing since 2000 [3], and by 2017, cancer became the country’s leading cause of death, constituting 26.1% of all deaths in China [4]. In response, the Chinese government has given increasing priority to researching, developing, and delivering effective cancer drugs.

Clinical trials for anti-cancer investigational new drugs (INDs) began to take place in China as early as 1960 and have developed rapidly since 2008 [5]. Research on cancer drugs has been continuously supported since 2009 by the Chinese Major New Drug Innovation Program [6]. Between 2009 and 2018, the number of cancer clinical drug trials in China increased at an average annual rate of 33% [6]. In response, since 2015, the Chinese government has issued a series of regulatory policies for promoting and developing innovative drugs and clinical trials [7]^.^ A total of 2602 clinical trials, mainly for anti-tumor drugs, were registered in 2020 [8], and the annual growth rate of China’s cancer clinical trials in 2020 was 52.3% [9]. Despite the increasing number of trials, a significant but understudied barrier to wider implementation of cancer clinical drug trials in China is the complexity of the informed consent process [10].

Informed consent is the application of the ethical principle of respect for persons [11] or respect for autonomy [12]. In clinical practice, the physician must obtain the patient’s voluntary informed consent prior to any medical care provided, ensuring that the patient receives and understands the information needed to make an independent, informed decision about the proposed care [13]. Relatedly, informed consent is the cornerstone of protection of human subjects in clinical trials [14]. Since 1945, international guidelines for responsible conduct of human experimentation in medical research have been established and built upon. The definition of informed consent has evolved as the best known of these codes emerged, including the Nuremberg Code of 1947 (which states that voluntary consent of the human subject is absolutely essential) [15], the Helsinki Declaration of 1964 (which states that clinical research on a human being cannot be undertaken without their free consent after they have been informed) [16], and the Belmont Report of 1979 (which outlines three elements of the consent process: information, comprehension, and voluntariness) [11, 17].

In addition to following international guidance on informed consent, Chinese laws and regulations for clinical research require researchers to obtain informed consent for studies [18]. In 2003, China issued its first “Good Clinical Practice for Drugs” (GCP) guidelines, which clearly emphasize ethics review committees and informed consent as the main measures to protect the rights and interests of study participants [19]. The revised GCP in 2020 established stricter and more detailed regulations on the content and signature process of informed consent forms (ICFs) [20]. And since 2019, a number of Chinese laws, including the Basic Medical and Health Care and Health Promotion Law, the Common Law, and the Drug Administration Law, have begun to officially require investigators conducting drug clinical trials and other medical research to obtain informed consent from participants [21].

Obtaining informed consent in cancer clinical drug trials involves unique ethical complications. Participants in these trials are often especially vulnerable — the majority being patients with advanced cancer who have no other therapeutic options, fairly low survival rates, and short expected remaining life spans [22, 23]. High costs associated with cancer treatment are known to impose significant economic burdens on participants and their families [23]. Furthermore, patients with cancer in China frequently have limited to no awareness of their diagnosis, due to a common practice of family members communicating directly with clinicians on the patient’s behalf [24].

The concept of informed consent emerged in the aforementioned international guidelines in a context largely shaped by Western liberal individualism [25] — a context in which ethics of clinical practice and scientific research involving human subjects have undergone a significant shift over the last few decades from a paternalistic model to a patient-centered approach. By contrast, Chinese traditional culture stemming from Confucianism stresses an individual’s moral obligations to the family or clan [26]. Some Chinese scholars have highlighted aspects of Confucianism and Chinese traditional medical culture that also in fact emphasize truthfulness to individuals [27]. However, due to a mainstream understanding of family-oriented values from Confucianism and traditional views on death as a taboo topic, family members in China often conceal diagnostic and prognostic information from cancer patients, in an effort to protect patients from worry and despair. Despite ongoing changes to Chinese law emphasizing the need to inform patients directly of medical information, and despite an increasing proportion of cancer patients who report they would want to be informed, oncology clinicians still tend to defer to families who prefer concealing information from patients [24, 28, 29]. It has not yet been widely studied how this practice may have specific implications for informed consent in cancer clinical drug trials in China.

In light of changing clinical trials regulations and cultural factors concerning disclosure of information to cancer patients in China, there is a particular need for research to examine informed consent in cancer clinical drug trials. This narrative review study aims to describe the current situation and identify challenges affecting informed consent in clinical trials of cancer drugs among adult patients in China since 2000.

## Methods

### Search strategy

Publications from 2000 to 2020 were comprehensively searched. A literature search was conducted in the following digital databases: Web of Science (WOS), PubMed, Scopus, EMBASE, and the Cochrane Library databases, China National Knowledge Infrastructure (CNKI), China Biomedical Literature Database on Disc (CBMdisc), Chinese Scientific Journals Fulltext Database (CQVIP), and WANFANG Data, the latter four of which are prominent digital databases in China. We used the following search words: cancer/tumor/oncology/neoplasm, informed consent, clinical trial, ethic/ethics/ethical, China/Chinese. We used the following search terms: (1) (“cancer” or “tumor” or “oncology” or “neoplasm”) AND “informed consent” AND “clinical trial” AND (“China” or “Chinese”); (2) (“cancer” or “tumor” or “oncology” or “neoplasm”) AND “informed consent” AND “clinical trial” AND (“ethic” or “ethics” or “ethical”) AND (“China” or “Chinese”). Search terms were different according to different databases. Specific strategies for each database are presented in Additional file 1. We conducted additional review by hand-searching for other relevant publications cited in the reference section of the included publications.

### Selection criteria and screening

We included both original studies and reviews related to the current situation or challenges affecting informed consent in clinical trials of cancer drugs among adult patients in China. Publications were excluded if they were (1) correspondence, editorials, or conference abstracts; (2) studies not written in Chinese or English; (3) studies not available in full text; (4) studies on informed consent in clinical trials among children or adolescents; (5) clinical trials of drugs that did not specify a target disease; (6) studies exploring how informed consent should be implemented; (7) clinical trials of medical instruments or new technologies rather than drugs, or clinical trials conducted outside China; or (8) studies on ethical issues in targeted clinical trials for cancer drugs that did not mention informed consent.

All publications identified according to these criteria went through title and abstract screening and then full-text screening, with both conducted independently by authors WZ and XL. We resolved disagreements by consensus during group discussions among three authors (XL, XRL, XMW).

### Data extraction

**Fig. 1:**
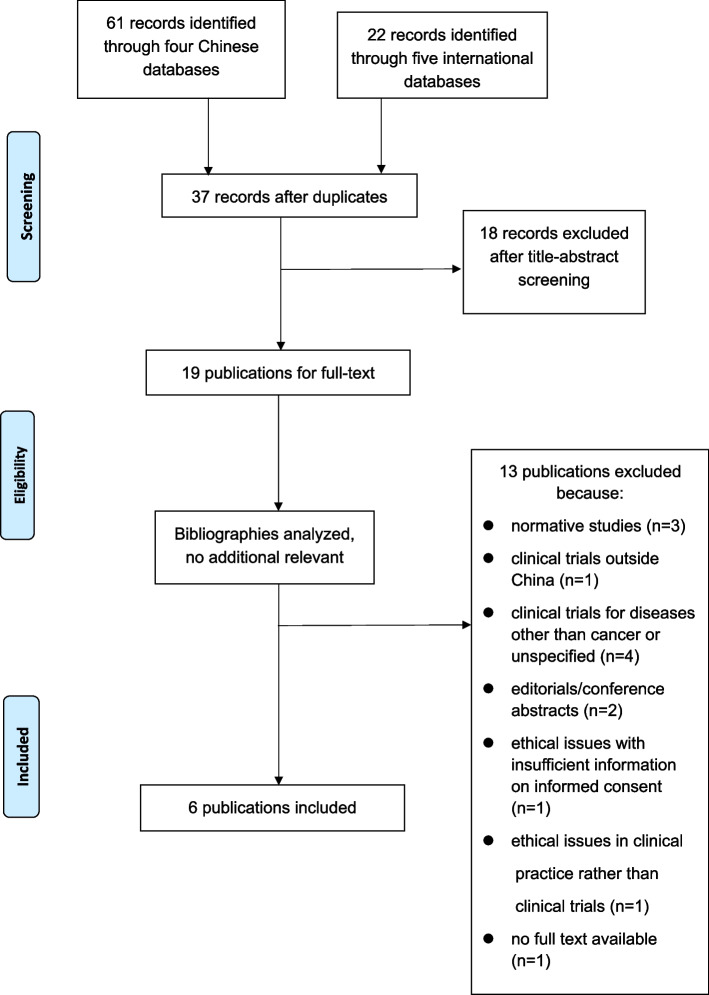
Flow diagram for the selection of studies

From each included publication, we extracted (1) the year of publication; (2) first author information, including names and institutions; (3) study design; (4) clinical trial phase number or other classification; and (5) qualitative or quantitative data on informed consent, including but not limited to ICFs; implementation procedures; and knowledge, experience, and satisfaction among researchers or participants. As our review was restricted by the limited number of eligible publications, only six publications were included in the final analysis (see Fig. 1).

### Review and thematic analysis

Thematic analysis focuses on identifying themes and the relationships between themes within a dataset [30]. Following the procedure outlined by Braun and Clarke [31], two authors (YQZ and XMW) performed thematic analysis. First, significant phrases and sections of the publications included in this review were coded inductively with reference to the research question; these initial codes were then sorted and clustered into categories, which enabled identification of themes derived from the data and centered on the research question of challenges in informed consent in cancer clinical drug trials. The themes were reviewed and refined iteratively and reflexively, until they were distinctive and coherent [31]. Discrepancies in the opinions of the two coders (YQZ and XMW) were resolved through discussion. If agreement was not reached, a third author (JH) was consulted to reach consensus. The NVivo software (Version 11) was used for the organization of data and thematic analysis.

### GRADE-CERQual assessment

We used the Grading of Recommendations Assessment, Development and Evaluation Confidence in the Evidence from Reviews of Qualitative Research (GRADE-CERQual) approach [32] to evaluate the publications included in this review. We evaluated our findings from the six publications based on the four criteria outlined by GRADE-CERQual: methodological limitations of included studies supporting a review finding, the relevance of included studies to the review question, the coherence of the review finding, and the adequacy of the data contributing to a review finding (Table 1). All findings started as high confidence, and we then downgraded the findings if we had important concerns regarding any of the GRADE-CERQual components.

**Table 1:** Results of GRADE-CERQual assessment

**Review findings**	**Studies contributing to the review findings**	**Methodological limitations**	**Relevance**	**Coherence**	**Adequacy**	**Overall assessment of confidence**	**Explanation of judgment**
In practice, treatment alternatives, side effects, and toxicity of drugs being researched were often only partially disclosed or entirely undisclosed to patients participating in clinical trials	[33–36]	Moderate methodological limitations (two studies with minor and two studies with no methodological limitations)	No or very minor concerns about relevance	No or very minor concerns about coherence	Minor concerns about adequacy (four studies that together offered moderately rich data overall)	Moderate	Finding graded as moderate because of concerns about methodological quality and adequacy of contributing papers
Cancer patients reported difficulty in understanding the meaning of key terms in clinical trials, and their knowledge regarding their rights and obligations during a clinical trial was insufficient	[33–35, 37]	Minor methodological limitations (one study with minor and three studies with no methodological limitations)	No or very minor concerns about relevance	No or very minor concerns about coherence	Minor concerns about adequacy (four studies that together offered moderately rich data overall)	High	Finding graded as high because of only minor concerns about methodological quality
Investigators exaggerated the potential therapeutic effect of clinical trials, understated the potential harms or side effects, or blurred the distinctions between treatments and clinical trials to strengthen the subject’s voluntariness	[33, 38]	Minor methodological limitations (one study with minor and one study with no methodological limitations)	No or very minor concerns about relevance	Minor concerns about coherence (one study with minor and one study with no concerns about coherence)	Minor concerns about adequacy (two studies that together offered moderately rich data overall)	Moderate	Finding downgraded because of concerns about methodological quality and adequacy of contributing papers
Investigators valued the preferences of family members more than patients and family members’ informed consent was mistakenly considered to be an adequate replacement for patients’ informed consent	[33, 35, 36, 38]	Moderate methodological limitations (two studies with minor and two studies with no methodological limitations)	No or very minor concerns about relevance	Minor concerns about coherence (one study with minor and three studies with no concerns about coherence)	Minor concerns about adequacy (three studies that together offered pretty rich data overall)	Moderate	Finding downgraded because of concerns about methodological quality and adequacy of contributing papers
There were common errors related to procedures, including: both clinical investigators and patients sometimes failed to sign consent forms after patients orally agreed to participate and many patients and even clinical researchers did not know that patients should receive duplicate copies of signed ICFs	[33, 35, 37]	Minor methodological limitations (one study with minor and two studies with no methodological limitations)	No or very minor concerns about relevance	No or very minor concerns about coherence	Minor concerns about adequacy (four studies that together offered pretty rich data overall)	High	Finding graded as high because of only minor concerns about methodological quality

## Results

Among the 37 publications initially identified (Additional file 2), only six met selection criteria. Review of the references cited by the six eligible studies did not result in identifying additional eligible studies (Fig. 1). The publications included for final analysis are listed in Table 2. All six studies were published in Chinese journals, and the publication years of five out of the six studies were 2015 or later. The authors of the six studies were all from clinical departments or ethics review committees at five hospitals in China. All of the included publications were descriptive studies, four of which provided a general overview of current implementation or challenges related to informed consent in cancer clinical drug trials based on the literature or the authors’ own experiences. The other two provided results based on questionnaires completed by cancer patients. Based on thematic analysis, five main themes emerged regarding challenges related to informed consent: information disclosure, patient understanding, voluntariness, authorization, and procedural issues.

**Table 2:** Included publications

**Title**	**Author**	**Institutions**	**Publication year**	**Study type**	**Main concepts**
Ethical Issues of Illiterate Patients in Clinical Trials of Anti-cancer Drugs	Hong, D., et al. [38]	Zhejiang Cancer Hospital	2015	Descriptive; narrative based on literature and author experience	Ethical issues, including informed consent among illiterate patients
Analysis and Countermeasures of Common Ethical Issues in the Medical Clinical Trial Implementation in Oncology Department	Li, A.M., et al. [35]	Department of Oncology, the First Affiliated Hospital of Zhengzhou University	2016	Descriptive; narrative based on literature and author experience	Ethical issues, including informed consent implementation
The Patient’ s Informed Consent in Clinical Trial of Anti-tumor Medicine Research	Jiang, K., & Zhang, Y. [33]	Department of Oncology, The Second Affiliated Hospital of Dalian Medical University	2017	Descriptive; narrative based on literature and author experience	Problems existing in the process of informed consent
Investigation and Thinking of Tumor Patients’ Understanding of Clinical Research Ethics	Wei, Y., et al. [37]	Department of Oncology, Radiotherapy and Chemotherapy, Zhongnan Hospital, Wuhan University	2017	Descriptive; cross-sectional survey; consecutive sampling in one hospital; 81 cancer patients but non-participants of clinical trials	Patients’ knowledge and attitudes toward clinical trials and related ethical issues, including informed consent
Specialty and Related Medical Ethical Issues in Clinical Trials of Anticancer Drugs	Zhang, L., et al. [36]	Medical Ethics Committee, Peking University Cancer Hospital & Institute	2017	Descriptive; narrative based on literature and author experience	Ethical issues, including informed consent implementation
Investigation on the quality of informed consent of subjects in clinical trials of anti-tumor drugs	Wu, M., Li,D., & Liu, X.H. [34]	Anesthesiology Department, National Drug Clinical Trial Center Office, Peking University Cancer Hospital & Institute	2019	Descriptive; cross-sectional survey; consecutive sampling in one hospital; 170 cancer patients and also participants in clinical trials of all phases	Quality of informed consent in clinical trials

### Information disclosure

In practice, treatment alternatives, side effects, toxicity of drugs being researched, and participant rights and responsibilities were often only partially disclosed or entirely undisclosed to patients participating in clinical trials [33–36]. In a survey on quality of informed consent in cancer clinical drug trials, 55.9% of 170 participants endorsed the statement: “My doctors did not offer any alternatives besides treatment in a clinical trial” [34]. And 41.2% reported not being sure whether doctors had offered information about alternative treatments [34]. Another common problem noted by authors was that investigators used complicated technical terms without adequate explanation when disclosing information or when providing ICFs [33].

### Patient understanding: quality of information disclosure

Due to the aforementioned challenges related to information disclosure, therapeutic misconception (failing to distinguish the difference in purpose between research and clinical medicine) and therapeutic misestimation (misperceiving the level of risk and chance of benefit in trials) were both prevalent [39]. Many participants in clinical trials believed that the drug or treatment being researched was the only effective or the best one for their cancer [33]. In terms of therapeutic misconception, results of a survey showed that over 70% of participants in cancer drug trials endorsed the statement: “Treatment regimens studied in trials have been proven to be the best for my tumor.” and 91.2% of participants mistook clinical trials as the standard-of-care treatment [34]. In terms of therapeutic misestimation, 80% of participants surveyed mistakenly believed that drugs in clinical trials would not cause severe side effects [34]. Without sufficient explanation from professionals, cancer patients also reported difficulty in understanding the meaning of key terms in clinical trials, such as “randomization” and “placebos” [33]. In addition, patients’ knowledge regarding their rights and responsibilities during a clinical trial was insufficient [35]. For example, less than 60% of patients with cancer were aware of their right to withdraw from a clinical trial [37].

### Voluntariness

Valid informed consent requires voluntariness, which refers to freedom from coercion or undue influence [11]. The studies reviewed did not suggest that potential trial participants often experienced coercion, meaning a direct threat of harm if they were to choose not to participate. However, undue influence was observed in the form of individuals in authority urging a certain course of action, direct pressure from close or authoritative others to participate, or strong emphasis on financial incentives [11]. Investigators were prone to tendentious explanations of trials; one author reported that investigators often “induced participation” among patients in that they “overstated the efficacy of trial protocols or understated known toxic side effects” [33]. One study reported more overt pressure on patients by clinicians or family members to join clinical trials and noted based on observation that patients with lower education level may be more vulnerable to such pressure [38]. This same author suggested that clinicians often emphasized the reduction or waiving of medical fees to recruit patients for clinical trials, thereby putting undue emphasis on financial incentives to participate [38].

### Authorization

Previous research has shown that oncology clinicians in China communicate primarily with family members, and many family members partially or completely conceal information from cancer patients [40]. Relatedly, for certain treatment decisions in cancer clinical drug trials, investigators both “paid more attention to communication with family members” than patients and “paid more attention to family members’ choices” — which raises ethical questions about the extent to which patients’ informed consent is currently being obtained [33, 36]. Chinese law allows for proxy consent to undergo clinical procedures or participate in clinical trials only when the patient is in a coma or in some other condition such that they lack capacity to make independent decisions [24]. In some cases that did not meet this criteria, family members’ informed consent was mistakenly considered to be an adequate replacement for patients’ informed consent, especially when patients were illiterate [35, 38].

### Procedural issues of informed consent

In the practice of informed consent in cancer clinical drug trials, there were common errors related to procedures, including (1) both clinical investigators and patients sometimes failed to sign consent forms after patients orally agreed to participate [33, 35], (2) some signed ICFs were missing signature dates [35], and (3) many patients and even clinical researchers did not know that patients should receive duplicate copies of signed ICFs [35, 37]; therefore, it was commonly found that “the participant did not get a copy after signing the informed consent” [33].

## Discussion

Cancer is a major public health problem in China, and the number of cancer clinical drug trials is increasing rapidly. This paper provides the first attempt to comprehensively review all relevant studies on informed consent in cancer clinical drug trials in China. Our results show that there is limited research on this topic to-date, despite its public health importance. In this review, we identified challenges in voluntariness and authorization, raising questions as to whether the full consent of participants in cancer clinical drug trials is being obtained in practice. We also identified challenges in information disclosure and patient understanding, raising questions as to whether consent, to the extent that is being obtained, is adequately informed. Lastly, our review highlighted procedural challenges, raising questions as to whether consent is being documented thoroughly enough for regulatory oversight. This discussion will address each set of challenges by providing context and implications for future research and practice.

Findings from this study suggest that undue influence from clinicians, investigators, and family members may in some cases compromise the voluntariness of trial participants’ consent. The study population involved in cancer clinical drug trials — often advanced cancer patients — may be especially vulnerable to undue influence to enroll in oncology-related protocols, due to therapeutic optimism [41]. For example, research shows that advanced cancer patients frequently overestimate life expectancy or misperceive palliative chemotherapy as a curative treatment [42]. Moreover, a US study found that patient-participants report a high degree of reliance on information from and trust in clinicians regarding whether to enroll in research [43]. Clinicians in the same study stressed the need to separate clinical from research consent, as a method of moderating perceived influence on the patient decision of whether to enroll [43]. Together, these findings point to the particular importance of clinician-investigators taking steps to prevent undue influence in cancer trials, including adopting a more in-depth, collaborative exploration of the risks and benefits of trial arms with patients and emphasizing voluntariness [44].

As for the issue identified in this review of family members giving authorization for patients to participate in trials, it is important first to recognize that literature in various countries views a patient’s decision to participate in a clinical trial as one that frequently involves his or her family members and/or caregivers. However, the ethical principle of patient autonomy suggests that researchers should ensure patients make the final decision about whether to participate, when resolving conflicts between the interests of patients and those of their relatives [23]. Chinese law concerning informed consent has increasingly moved toward recognition of patient autonomy over the last three decades. From 1994 to 2002, the law stipulated that medical exams, procedures, and treatments could only be carried out after obtaining the consent of both the patient and their close relatives [45]. In 2002, the Regulations on Handling Medical Accidents began to shift this rule, stating that medical information must be provided directly to the patient and only if it was impossible or inappropriate to do so could the family receive information on the patient’s behalf [45]. After these regulations were followed by the 2009 Tort Liability Law and the Basic Norms of the Documentation of the Medical Record in 2010, it became a legal rule that only the patient themselves could provide informed consent for medical care, except for situations in which this was “impossible” (a term which left clinicians some room for interpretation) — in which case family members could consent on the patient’s behalf [45].

Despite changes to Chinese law, a 2018 review suggested that in practice, clinicians in China in the 2010s still largely communicated first with family members and in fact obtained written consent more often from family members than directly from patients [45]. Later in 2018, the “Regulations on the Prevention and Handling of Medical Disputes” made the requirement to obtain the individual patient’s informed consent for clinical care and clinical trials even more clear. These regulations specified that close relatives should only provide proxy consent for patients in the case of a coma or another medical condition that either made it unsuitable to explain information to the patient or made the patient unable to make decisions [24]. In line with the 2018 review showing a gap between legal changes and changes in clinical practice, findings from the present review suggest that clinicians and investigators in cancer clinical drug trials frequently obtain proxy consent from family members in situations that do not meet legal criteria for bypassing the consent of the patient. These findings on issues of authorization may be considered the downstream effects of our findings on information disclosure.

Findings from the present review suggesting that patients were often partially or fully uninformed of key information about risks, side effects, and alternatives of cancer clinical drug trials aligns with existing research on cancer information disclosure in China. Research suggests that although as many as 98% of surveyed cancer patients in China believe patients should be informed of a cancer diagnosis [46], physicians conceal cancer-related information from patients in 35.8–50.3% of cases [47] — largely because families ask them to protect the patient from the psychological impact of knowing [48]. Findings from this review suggest that the tendency to fully or partially conceal information about cancer and treatment options from patients may also occur in the context of cancer clinical drug trials.

While withholding information about cancer and other serious illnesses from patients is standard practice in China, there is currently a lively debate in Chinese bioethics scholarship as to whether it can truly be said to represent traditional Chinese cultural values. Some scholars argue that the Confucian value of mutual interdependence in the family means that clinicians should recognize the family’s autonomy to protect the patient’s best interests [49]. But others have noted that Confucianism also places a high value on truthfulness and suggested that this should motivate clinicians to inform the individual patient [27]. Moreover, arguments from transcultural bioethics have highlighted that present-day, contemporary Chinese culture integrates a spectrum of values and beliefs, including ancient and modern, Western and Eastern — and that this plurality of experiences and moralities should be taken into account in contemporary Chinese bioethics [50].

On a practical note, many patients who are not directly informed of a serious illness come to infer their diagnosis on their own, based on clues in their care environment, information overheard, or other means; a 2021 survey of Chinese cancer patients showed that 19.7% inferred their diagnosis even when not directly told [51]. Patients who know the truth may then pretend not to know, out of concern for their loved ones who were trying to show them care by concealing the truth [50]. In light of the added suffering that patients may experience in silently knowing the truth, it could be argued that working toward more transparent communication in the clinician-family-patient relationship may actually enhance the family’s ability to protect and care for the patient, in the end [27].

In order to move toward more transparent communication and patient-centered informed consent, greater attention is needed toward the rebuilding of clinician-family-patient trust [52]. Both medical malpractice claims and violence against physicians are occurring at increasing rates in China [24]. Previous research by the present authors found that fear of retaliation from family members was a main reason that physicians chose not to inform cancer patients of their diagnosis [24] — a key first step to patient involvement in the informed consent process for cancer treatment, including clinical trials. Chinese physicians in clinical settings deciding whether to inform patients of a cancer diagnosis and treatment options have also reported facing ethical tension between the patient’s individual right to know their diagnosis and decide on treatments and the family’s interest in protecting the patient [53]. To inform the improvement of medical trust in cancer clinical trials, future research should examine whether this same ethical tension may be at play in cancer trials, or whether unique challenges exist in this context as to why clinician-investigators may withhold or underemphasize key information about trials.

The present review also found that even when ICFs are used to disclose a certain amount of information, participants in cancer clinical drug trials in China frequently maintained misconceptions about information such as risks, rights as a participant, and alternative treatment options. This aligns with previous studies showing that Chinese clinical trial ICFs had lower readability and less complete content than clinical trial ICFs from other countries [54]. However, research also shows that in countries around the world, misconceptions about cancer clinical trials remain common [55].— a problem exacerbated by the lengthiness and complexity of ICFs [56]. Participants’ lack of adequately informed consent may lead to less satisfaction with their decision to enroll or regret about having enrolled [57]. It is ethically imperative that future research both in China and at an international level continues to develop strategies to increase participants’ understanding of ICF content [58].

At present, an increasing number of voices internationally and in China are calling for patients’ perspectives to be incorporated into the design and conduct of clinical trials, to align both information in the consent process and trial procedures more closely with the needs of patients [59]. Expert consensus recommendations in other countries have emphasized the need to include patients in the design of the informed consent process, including consent documents [6]. In line with international efforts, the Drug Review Center of the State Food and Drug Administration in China issued a 2022 circular on “Guidelines for the General Consideration of Organizing Patients to participate in Drug Research and Development (Trial Implementation)” [60]. The guidelines state that in the process of drug development (including clinical trials), emphasis should be placed on listening to and focusing on the perspective of the patient, as well as the patient’s family members, guardians, caregivers, and patient organizations or advocacy groups [60]. Future research should evaluate how effectively these guidelines are applied in practice.

Findings from this review also indicated that procedural issues are common in cancer clinical drug trials in China, including missing signatures on ICFs and a lack of distribution of copies of ICFs to participants. Institutional Review Boards (IRBs) have the responsibility to ensure ethical protection of human research participants, including review of research protocols, informed consent processes, and other documents and procedures [41]. Institutional Review Boards (IRBs) have been increasing in number in China since the 1990s, along with regulations to support the development of IRBs [61]. According to Chinese law as of 2020, all research, including cancer clinical drug trials, must be reviewed by IRBs [61]. However, given the relatively late beginning of the development of IRBs in China, quality assurance of IRB review and oversight of research are still at an early stage [61]. Findings from this study regarding procedural issues of informed consent add to existing evidence showing the need for improved quality assurance of IRBs in China. At present, only a few Chinese research institutions have obtained accreditation for Human Research Protection Programs (HRPPs), which oversee quality and efficiency of IRBs, and the continuous improvement and training of IRBs [61]. Further establishment of HRPPs in China may help to increase the standardization of informed consent documentation. Particularly in the context of cancer clinical trials, special considerations for informed consent are more likely to arise, including those related to secondary use of biological samples, big data research for group health, and use of electronic informed consent [62]. As HRPPs and other regulatory mechanisms for IRB oversight continue to develop in China, it will be necessary to address training needs for clinicians and investigators involved in these and other research procedures.

## Limitations

Due to the limited number of studies currently published on informed consent in cancer clinical drug trials in China, our final analysis for this review included six studies — a smaller number than would allow for a more full, comparative review of 20 years of literature. The publications analyzed constituted relatively little original data, as 4 of the 6 used expert opinions as the main data source. Research based on expert opinions, without additional quantitative data collected via validated instruments, carries the risk of reporting bias, because the information is based on the authors’ own experiences and cannot be generalized to understand how widely and acutely the proposed challenges related to informed consent affect China’s cancer clinical drug trials. This limitation also implies limited potential to compare the results of this review with research in other countries. The two publications included in our review that were based on cross-sectional surveys, rather than expert opinion, had samples that were small in size and recruited from single hospitals, limiting the representativeness of their results.

Our analysis also indicated existing studies on this topic in China discussed issues of informed consent without considering different phases of clinical trials or different types of cancers, which further limits generalizability. It is worth noting that studies outside China have found different characteristics among different subgroups of patients, regarding informed consent in clinical trials. For example, participants in phase I clinical trials are more likely to have misconceptions related to therapeutic optimism and less likely to understand the purpose of clinical trials than participants in clinical trials of later phases [62].

## Conclusion

Based on a thorough analysis of existing publications, this review identified five main challenges related to various aspects of informed consent in cancer clinical trials in China: information disclosure, patient understanding, voluntariness, authorization, and procedural issues. Greater effort should be devoted to improving the readability, content, and explanation of ICFs to ensure the rights of potential participants. Furthermore, despite the fact that the number of cancer clinical drug trials is growing rapidly in China, findings from this review suggest that only a limited number of empirically based research studies on informed consent in cancer clinical drug trials in China have been conducted to date. Efforts toward improving informed consent practice, in the form of guidelines or further regulations in China, should draw on both experience from other countries and high-quality local evidence from future studies.

## Supplementary Information

**Additional file 1.** Specific strategies

**Additional file 2.** The publication list for title and abstract screening

## Data Availability

Not applicable
