# Supplementary material for: Informed consent in cancer clinical drug trials in China: a narrative literature review of the past 20 years
Source: Trials. 2023 Jul 7;24:445. doi: 10.1186/s13063-023-07482-y (PMC10327323; doi:10.1186/s13063-023-07482-y)
Supplement: Supplementary file 1 — Additional file 1. Specific strategies [file 13063_2023_7482_MOESM1_ESM.pdf]

Appendix 1 Specific strategies

Wangfang Data, CQVIP & CMBdisc

1. (informed consent)ti.

2. (clinical trial)all.

3. (cancer or oncology or neoplasm or tumor or carcinoma)all.

4. (ethic or ethics or ethical)ti.

5. (informed consent)all.

6. 1 and 2 and 3(publication date from 2000/1/1 to 2020/12/31)

7. 2 and 3 and 4 and 5(publication date from 2000/1/1 to 2020/12/31)

8. 6 or 7

CNKI

1. (informed consent)ti.

2. (clinical trial)mh.

3. (cancer or oncology or neoplasm or tumor or carcinoma)mh.

4. (ethic or ethics or ethical)ti.

5. (informed consent)mh.

6. 1 and 2 and 3(publication date from 2000/1/1 to 2020/12/31)

7. 2 and 3 and 4 and 5 (publication date from 2000/1/1 to 2020/12/31)

8. 6 or 7

WOS

1.(informed consent or exp informed consent or consent or consent* or

informed adj3 consent or informed decision or informed consent form or informed consent procedure or informed consent process or informed consent right or informed consent system or informed consent statement or informed choice or informed assent or assent or assent*)ti.

2.(clinical trial)tw.

3.(cancer or oncology or neoplasm or tumor or carcinoma)tw.

4.(China or Chinese)tw.

5.(ethic or ethics or ethical)ti.

6.(informed consent or exp informed consent or consent or consent* or informed adj3 consent or informed decision or informed consent form or informed consent procedure or informed consent process or informed consent right or informed consent system or informed consent statement or informed choice or informed assent or assent or assent*)tw.

7. 1 and 2 and 3 and 4(publication date from 2000/1/1 to 2020/12/31)

8. 2 and 3 and 4 and 5 and 6(publication date from 2000/1/1 to 2020/12/31)

9. 7 or 8

PubMed & Cochrane Library:

1.(informed consent or exp informed consent or consent or consent* or informed adj3 consent or informed decision or informed consent form or informed consent procedure or informed consent process or informed consent right or informed consent system or informed consent statement or informed

choice or informed assent or assent or assent*)ti.

2.(clinical trial)all.

3.(cancer or oncology or neoplasm or tumor or carcinoma)all.

4.(China or Chinese)all.

5.(ethic or ethics or ethical)ti.

6.(informed consent or exp informed consent or consent or consent* or informed adj3 consent or informed decision or informed consent form or informed consent procedure or informed consent process or informed consent right or informed consent system or informed consent statement or informed choice or informed assent or assent or assent*)all.

7. 1 and 2 and 3 and 4(publication date from 2000/1/1 to 2020/12/31)

8. 2 and 3 and 4 and 5 and 6(publication date from 2000/1/1 to 2020/12/31)

9. 7 and 8

EMBASE

1.(informed consent or exp informed consent or consent or consent* or informed adj3 consent or informed decision or informed consent form or informed consent procedure or informed consent process or informed consent right or informed consent system or informed consent statement or informed choice or informed assent or assent or assent*)ti.

2.(clinical trial)tw.

3.(cancer or oncology or neoplasm or tumor or carcinoma)tw.

4.(China or Chinese)tw.

5.(ethic or ethics or ethical)ti.

6.(informed consent or exp informed consent or consent or consent* or informed adj3 consent or informed decision or informed consent form or informed consent procedure or informed consent process or informed consent right or informed consent system or informed consent statement or informed choice or informed assent or assent or assent*)tw.

7. 1 and 2 and 3 and 4(publication date from 2000/1/1 to 2020/12/31)

8. 2 and 3 and 4 and 5 and 6(publication date from 2000/1/1 to 2020/12/31)

9. 7 and 8

Scopus

1.(informed consent or exp informed consent or consent or consent* or informed adj3 consent or informed decision or informed consent form or informed consent procedure or informed consent process or informed consent right or informed consent system or informed consent statement or informed choice or informed assent or assent or assent*)ti.

2.(clinical trial)ti,ab,kw.

3.(cancer or oncology or neoplasm or tumor or carcinoma)ti,ab,kw.

4.(China or Chinese)ti,ab,kw.

5.(ethic or ethics or ethical)ti.

6.(informed consent or exp informed consent or consent or consent* or

informed adj3 consent or informed decision or informed consent form or informed consent procedure or informed consent process or informed consent right or informed consent system or informed consent statement or informed choice or informed assent or assent or assent*)ti,ab,kw.

7. 1 and 2 and 3 and 4(publication date from 2000/1/1 to 2020/12/31)

8. 2 and 3 and 4 and 5 and 6(publication date from 2000/1/1 to 2020/12/31)

9. 7 and 8
