# Supplementary material for: Informed consent in cancer clinical drug trials in China: a narrative literature review of the past 20 years
Source: Trials. 2023 Jul 7;24:445. doi: 10.1186/s13063-023-07482-y (PMC10327323; doi:10.1186/s13063-023-07482-y)
Supplement: Supplementary file 2 — Additional file 2. The publication list for title and abstract screening [file 13063_2023_7482_MOESM2_ESM.pdf]

Appendix 2 The publication list for title and abstract screening

1. Hamilton,A.(2002).Ethical issues surrounding the conduct of off-shore clinical research. J Clin Oncol. 20(18),3934-3936.

2. Savitz,S.I.,Rivlin,M.M.,&Savitz,M.H.(2002).The ethics of prophylactic antibiotics for neurosurgical procedures. J Med Ethics. 28(6),358-363.

3. Li,S,T.(2002).Localization of informed consent form in the international multi-centered clinical trial.Chinese Journal of New Drugs.(06),430-431.

4. Mariner,W.K.(2003).Taking informed consent seriously in global HIV vaccine research. J Acquir Immune Defic Syndr.32(2),117-123.

5. Wang,J.M.,etal.(2003).Ethics in cancer research.Chinese Cancer.(06),4-6.

6. Lynöe, N., et al.(2004). Informed consent in China: quality of information provided to participants in a research project. Scand J Public Health. 32(6),472-5.

7. Li,S.T.(2005). Treat the subjects with a scientific and honest attitude-and discuss the problems in the informed consent form.Chinese Journal of New Drugs. (01),8-9.

8. Jiang,K.,&Zhang,Y.(2007).The Patient' s Informed Consent in Clinical Trial of Anti-tumor Medicine Research.Medicine and Philosophy(Clinical Decision Making Forum Edition).(11),68-69.

9. Wang,L.(2007).Communication: an important process of informed consent. Chinese prescription drugs.(09),43-44.

10. Huang,Y.P.,et al. (2008).Study on the effect of nursing notification and

informed consent system in preventing nurse-patient disputes in the oncology department.Chin J Prae Nurs.(19),67-68.

11. Benn,P.A.&Chapman,A.R.(2010).Ethical challenges in providing noninvasive prenatal diagnosis.Curr Opin Obstet Gynecol. 22(2),128-134.

12. Henderson,G.E.(2011). Is informed consent broken? Am J Med Sci. 342(4), 267-72.

13. Sugarman, J., et al.(2011).Are there adverse consequences of quizzing during informed consent for HIV research? J Med Ethics. 37(11),693-697.

14. Zhai,H.,Zhong,W.&Wu,Y.(2015).Research,evidence,and ethics: new technology or grey medicine. Ann Transl Med. 3(2),15.

15. Deng,D.Y.,Hu,D.H.&Liu,Y.S.(2015).Ethical Evaluation of Papers in Chinese Medical Journals.Research on Chinese Sci-tech Journals. 26(05),513-519.

16. Hong,D., et al.(2015).Ethics Issues of Illiterate Patients in Clinical Trails of Anti-cancer Drug.China Cancer.24(10),838-840.

17. Huang,J. & Wang,X.J. (2015). Medical Ethical Issues in the Clinical Trials of Anti-Cancer Drugs. China Cancer. 24(10), 834-837.

18. Ji,L. & Zhao,Q. (2015). Discussion on Problems and Countermeasures for Informed Consent in Drug Clinical Trials. Chinese Pharmaceutical Affairs. 29(04),412-416.

19. Zeng, L., et al.(2016). Do Chinese Researchers Conduct Ethical Research and Use Ethics Committee Review in Clinical Trials of Anti-Dementia Drugs?

An Analysis of Biomedical Publications Originating from China. J Alzheimers Dis.52(3), 813-23.

20. Cen,H.F. & Wang,Q.M.(2016). Thinking on the writing of informed consent for drug clinical trials. Medical Information. 29(31).

21. Zeng,L.F.,et al.(2016).Ethics and rights protection of vulnerable group in clinical trials of drugs for dementia.Chinese Journal of New Drugs. 25(24),2793-2798.

22. Li,A.M.,et al.(2016).Analysis and Countermeasures of Common Ethical Issues in the Medical Clinical Trial Implementation in Oncology Department.Chinese Medical Ethics.29(02),308-310.

23. Zhao,S.H.,et al.(2016).Analysis of problems in clinical informed consent.Chinese Journal of New Drugs.25(23),2692-2695.

24. Wang,W., et al. Ethical Review Issues and Countermeasures of Informed Consent Form for Drug Clinical Trials of Cancer Patients. (2017). The 19th Annual Academic Conference of the Medical Ethics Branch of the Chinese Medical Association and the International Forum on Medical Ethics.

25. Wei,Y., et al.(2017).Investigation and Thinking of Tumor Patients' Understanding of Clinical Research Ethics.Paper presented at the 19th Annual Academic Conference of the Medical Ethics Branch of the Chinese Medical Association and the International Forum on Medical Ethics.

26. Zhang,L.,et al.(2017).Specialty and Related Medical Ethical Issues in Clinical Trials of Anticancer Drugs.Cancer Res Prev Treat.44(07),506-508.

27. Liu,J., et al.(2018). Stem Cells Induced Differentiation for Reproductive Gamete and Concerning Ethical Consideration.China Science and Technology Forum. (03),152-158.
28. Liu,L.Y., Zhang,W.B. & Pu,C.(2018).Investigation on the Researcher'S Understanding of Informed Consent of Subjects in Clinical Trail.Medicine and Philosophy. 39(11),23-25.
29. Liu,D.,Zeng,S.Y. & Zhou,J.Y. (2019). Requirements of CAR-T Cell Immunotherapy on the Ethical Review.Chinese Medical Ethics. 32(01),59-62.
30. Wu,M.,Li,D.,&Liu,X.H.(2019).Investigation on the quality of informed consent of subjects in clinical trials of anti-tumor drugs.Chinese Journal of NewDrugs .28(24), 2981-2986.
31.Zhang,T.M. & Li,B.L.(2019). How to Effectively Sign Informed Consent In Drug Clinical Trials. Clinical Research. 27(05),191-193.
32. Li, J., et al.(2020).Effect of video-assisted education on informed consent and patient education for peripherally inserted central catheters: a randomized controlled trial. J Int Med Res.48(9), 300060520947915.
33. He,L.,et al. (2020). Discussion and Practice on Ethical Issues in Clinical Trials of Anti-tumor Drugs for the Chinese Children. Chinese Medical Ethics. 33(02),175-179.
34. Zhou,L.&Zhang,G.B.(2020).Analysis of ethical problems in clinical research of CAR-T cellular immunotherapy.Immunological Journal. 36(11),921-925.

35. Li,S.,et al.(2019). The protection of subjects in clinical trials of anticancer drugs-based on the perspective of ethical review. Proceedings of the 2019 Chinese Oncology Conference.

36. Editorial department.(2013).Explanation of Medical Ethics Requirements for Papers Submitted by this Journal.Chinese Journal of Oncology Surgery. 5(05),331.

37. Wang,H.,et al.(2004). Evaluation of the informed consent process in a randomized controlled trial in China: the Sino-U.S. NTD project. J Clin Ethics. 15(1):61-75.
